# Raised Intracellular Calcium Contributes to Ischemia-Induced Depression of Evoked Synaptic Transmission

**DOI:** 10.1371/journal.pone.0148110

**Published:** 2016-03-02

**Authors:** Shirin Jalini, Hui Ye, Alexander A. Tonkikh, Milton P. Charlton, Peter L. Carlen

**Affiliations:** 1 Division of Neurology, Department of Medicine, Queen’s University, Kingston, ON, Canada; 2 Institute of Medical Science, University of Toronto, Toronto, ON, Canada; 3 Krembil Research Institute, University Health Network, Toronto, ON, Canada; 4 Department of Biology, Loyola University Chicago, Chicago, IL, United States of America; 5 Department of Physiology, University of Toronto, Toronto, ON, Canada; Sackler Medical School, Tel Aviv University, ISRAEL

## Abstract

Oxygen-glucose deprivation (OGD) leads to depression of evoked synaptic transmission, for which the mechanisms remain unclear. We hypothesized that increased presynaptic [Ca^2+^]_i_ during transient OGD contributes to the depression of evoked field excitatory postsynaptic potentials (fEPSPs). Additionally, we hypothesized that increased buffering of intracellular calcium would shorten electrophysiological recovery after transient ischemia. Mouse hippocampal slices were exposed to 2 to 8 min of OGD. fEPSPs evoked by Schaffer collateral stimulation were recorded in the stratum radiatum, and whole cell current or voltage clamp recordings were performed in CA1 neurons. Transient ischemia led to increased presynaptic [Ca^2+^]_i,_ (shown by calcium imaging), increased spontaneous miniature EPSP/Cs, and depressed evoked fEPSPs, partially mediated by adenosine. Buffering of intracellular Ca^2+^ during OGD by membrane-permeant chelators (BAPTA-AM or EGTA-AM) partially prevented fEPSP depression and promoted faster electrophysiological recovery when the OGD challenge was stopped. The blocker of BK channels, charybdotoxin (ChTX), also prevented fEPSP depression, but did not accelerate post-ischemic recovery. These results suggest that OGD leads to elevated presynaptic [Ca^2+^]_i_, which reduces evoked transmitter release; this effect can be reversed by increased intracellular Ca^2+^ buffering which also speeds recovery.

## Introduction

Oxygen-glucose deprivation (OGD) is considered to be the major underlying pathophysiological mechanism in stroke, a major cause of death and disability in the general population [1]. Transient OGD is associated with clinically defined transient ischemic attacks, which are associated with reversible cerebral deficits. Upon exposure to OGD, the directly affected brain region rapidly loses function, which many attribute to synaptic dysfunction [2,3]. The CA1 region of the hippocampus is known to be quite sensitive to OGD [4] which causes quick and reversible effects on synaptic transmission onto CA1 pyramidal neurons, depressing both the field excitatory postsynaptic potential (fEPSP) and the population spike [5, 6]. The exact mechanisms that mediate these changes remain unclear and various theories, including presynaptic failure as well as postsynaptic failure, have been proposed.

Postsynaptically, anoxic depolarization of the postsynaptic membrane has been shown to reduce membrane excitability and contribute to failure of evoked transmission [7–9]. Structural changes in the postsynaptic density (PSD) after ischemia have also been reported and include NMDA receptor inactivation [10, 11], as well as loss of dendritic spines [12]. In CA3 neurons, it has been proposed that the depression of synaptic transmission is due to metabotropic glutamate receptor (mGluR) and adenosine-dependent removal of postsynaptic AMPA receptors [13], as well as activation of calcium-dependent downstream pathways. Cholesterol extraction from the lipid membrane by cyclodextrins has been shown to reduce neuronal excitability by disruption of NMDA and AMPA receptors that are localized to lipid rafts [14–16].

However, there is also much evidence supporting the notion that early synaptic failure in ischemia is a result of presynaptic malfunction and impaired transmitter release. Previous studies have shown that ischemia-induced increase in the concentrations of adenosine plays a major role. Adenosine is by-product of ATP-metabolism via catabolism by a variety of enzymes [17, 18]. It acts primarily on A1 receptors in the brain and attenuates presynaptic calcium currents through voltage-gated calcium channels (VGCC; [19, 20]), which subsequently depresses neurotransmission. Other presynaptic mechanisms include structural damage to the presynaptic apparatus, resulting in loss of synaptic buttons and projections [21, 22], as well as changes in intracellular calcium concentration [23–25].

Presynaptic transmitter release depends on the Ca^2+^ entry that occurs upon action potential (AP) invasion of the presynaptic membrane [26–27] and, owing to the 4th power dependence of transmitter release on intracellular [Ca^2^], even minor modulations of presynaptic Ca^2+^ can have dramatic effect on neurotransmitter release. Ischemia-associated rise in intracellular calcium is thought to occur through inflow from the extracellular environment, as well as release from internal stores. This has been thought to inactivate voltage-gated calcium channels (VGCCs), thus reducing transmitter release [28, 29]. Moreover, the increase of cytosolic Ca^2+^ that follows ischemia has many dysfunctional effects on the cell and is a crucial event leading to cell death [30–31].

In addition to VGCCs, large conductance Ca^2+^- activated K^+^ channels (BK channels), which are both voltage and calcium regulated, have been shown to play a key role in controlling presynaptic neurotransmitter release [32–35]. These channels are found throughout the vertebrate nervous system and are targeted to the active presynaptic zone of glutamatergic synapses [32, 36], in close proximity to VGCCs [37, 38]. AP-induced membrane depolarization and Ca^2+^entry through Ca^2+^channels activates BK channels, which contribute to termination of the AP, production of the fast after-hyperporlarization and shutting off of the calcium channel [39, 40]. Recent studies have implicated an important role for these channels in many neurological disorders, including fragile X syndrome, schizophrenia, autism and epilepsy [41–44]. However, very little work has been done on the role of these or other K^+^ channels during brain ischemia. Other presynaptic K^+^ channels that also play a role in modulating presynaptic depolarization include Kv1 channels [45–46], neuronal M-type K(+) channels [47], K_ATP_ channels [48] and Kv3 channels [49].

The objective of this study was to further investigate the role of raised presynaptic [Ca^2+^]_i_ during ischemia and the mechanisms by which it contributes to fEPSP attenuation. We hypothesized that increased presynaptic [Ca^2+^]_i_ during OGD contributes to the depression of evoked EPSPs and that this is partially mediated by the [Ca^2+^]_i_-mediated activation of BK channels. However, this decrease in neurotransmitter release also depresses normal synaptic functioning which, if prolonged, could impair functional recovery, Additionally we hypothesized that buffering [Ca^2+^]_i_ will diminish the pathological effects of OGD-mediated increased presynaptic [Ca^2+^]_i_, hastening the electrophysiological recovery of the tissue after transient ischemia.

## Material and Methods

### 1. Animals

B6C3F1 mice (4–9 weeks, Charles River) were used in this study. Experiments were done after protocols were approved by the Animal Care Committee at the University Health Network. Care was taken as to avoid unnecessary pain and suffering of the animals.

### 2. Tissue Preparation

Mice were anaesthetized with ketamine (IP, 10 mg/kg) and transcardial perfusion was performed with cold, oxygenated sucrose-based artificial cerebrospinal fluid (ACSF). The animal was decapitated and the brain was quickly removed and placed in ice-cold (2–5°C) sucrose-based ASCF for ~ 3–5 min. Sucrose-based ACSF contained (in mM): 210 sucrose, 26 NaHCO_3_, 2.5 KCl, 1 CaCl_2,_ 4 MgCl_2_, 1.25 NaH_2_PO_4_, and 10 glucose, and was continuously bubbled with 95% O_2_−5% CO_2._ This high Mg^2+^-low Na^2+^-containing ACSF was used only during tissue preparation to minimize dissection-induced damage, by reducing Na^+^-dependent toxicity [50] and has been shown to extend tissue viability [51]. The cerebellum was removed and the brain was bisected along the midsagittal line. The superior cortex was removed and the dorsal cortex was cut parallel to the longitudinal axis. Cyanoacrylate glue was then used to fix the brain, ventral side up, to an aluminum block. The block was secured at a 12° angle in a Vibratome (Series 1000, Technical Products International, St. Louis, MO) so that the caudal end of the brain faced the blade. Slices (400 μm) were incubated in room temperature ACSF for at least 1 hour before being transferred to the recording chamber. This ACSF, which was also used during perfusion of the slices while in the recording chamber, contained (mM): 123 NaCl, 26 NaHCO_3_, 2.5 KCl, 1.8 CaCl_2_, 0.9 MgCl_2_, 1.25 NaH_2_ PO_4_, and 10 glucose, and was continuously bubbled with 95% O_2_−5% CO_2._

Slices destined for pre-incubation with calcium chelators were incubated in normal ACSF for 30min before being transferred to solution containing chelator and probenecid (1 mM). Probenecid, an anion transport inhibitor, has been shown to accelerate and enhance the depression of synaptic transmission by BAPTA concentrations as low as 0.05 μM [52].

### 3. Extracellular Recordings

Once in the recording chamber, slices were perfused continuously with oxygenated ACSF (normal or + drug) at a rate of 15 ml/min. An automatic temperature control unit allowed a water bath underneath the recording chamber to be maintained at 36 ± 0.5°C, thus allowing the perfusing ACSF to be warmed to this set temperature. Additionally, warm, humidified 95% O_2_−5% CO_2_ gas was superfused over the slice and was switched to a 95% N_2_- 5% CO_2_ mixture during OGD episodes. This ensured that the slice had access only to the gas mixture and air was largely excluded.

A stimulating bipolar electrode (enamel-insulated nichrome wire, 125 μm diameter) stimulated the Schaffer collateral-commissural fibers for orthodromic activation of CA1 neurons. Extracellular fEPSPs were recorded by a borosilicate glass pipette filled with NaCl (150 mM) placed in stratum radiatum. Stimulation current of varied amplitude was given by a Grass S88 Stimulator (Grass Instruments, Quincy, MA). Signals were recorded, amplified, and filtered with an Axoclamp 2A amplifier in bridge mode (Axon Instruments, Foster city, CA) and acquisition of data was performed using pClamp version 6.0.3 software (Axon Instruments). Throughout the experiment, paired-pulse stimulation was administered at an interstimulus interval (ISI) of 50 ms.

OGD was induced by changing the ACSF aerated with 95% O_2_−5% CO_2_ to zero-glucose solution aerated with 95% N_2_- 5% CO_2._ This OGD-ACSF contained the same mixture as normal ACSF, except glucose was replaced with an equimolar amount of sucrose. Once response had stabilized, an input/output (I/O) curve was obtained by applying 15 pulses (0.1 ms) of 100 μA to 1500 μA in 100 μA steps. The stimulus that produced a response amplitude ~50–60% of maximum was selected as the “test intensity” for all subsequent procedures.

Prior to beginning experiments, responses were recorded for 10 min to ensure they were stable. Drug solutions, including chelators, were run for 30 min to allow for the drugs to take effect and for responses to stabilize. OGD was then administered for 2 to 8 min followed by recovery in the drug solution, and subsequent recovery in normal ACSF.

### 4. Intracellular Recordings

The recording chamber was mounted on a Zeiss Axioskop FS upright microscope (Neumann/Zeiss). Infrared differential interference contrast (IR-DIC) microscopy was used to visualize individual neurons and to guide the pipette for whole-cell patch clamp recording. Patch clamp electrodes were positioned onto the cell membrane under visual guidance using a motorized Newport XYZ translation stage.

Whole-cell recordings were performed using an Axoclamp 200B amplifier (Axon Instruments, Union City, CA, USA). Components of the patch pipette (intracellular) solution were (mM): 150 potassium gluconate, 2 Hepes, 0.1 EGTA (pH 7.25 and 280–290 mosmol l^–1^). Patch pipettes were pulled from borosilicate capillary tubing (World Precision Instruments, Sarasota, FL, USA) with a Narishige pipette puller (NG-811). Electrodes had tip resistances ranging from 4 to 6 MΩ when filled with solution. The resistance to ground of the whole-cell seal was 2–4 GΩ before breaking through the membrane and the series resistance was less than 20 MΩ. Pyramidal cells were recorded with whole-cell configuration in current clamp and voltage clamp modes. Data acquisition, storage and analyses were performed using pCLAMP software (version 9.2, Axon Instruments). Digitization was achieved using a 12-bit A/D board (Digidata 1200, Axon Instruments).

Spontaneous miniature EPSCs (mEPSCs) were detected using the event detection algorithm in Clampfit 9.0 (Axon Instruments). Briefly, a template was created by averaging multiple representative synaptic events. In some cases, more than one category of template was created to ensure reliable detection. The algorithm allowed the users to set a “threshold” for the reliable event detection while maintaining certain flexibility. A higher template match threshold ensures higher similarity between the template and the detected events, while a lower value increases the chance of false positives. In practice, we used the default value, 4, which provided a good balance. During event detection, experienced observers visually accepted the matched synaptic events and rejected abnormal ones in a few cases, which were likely due to noise. Using this method, over 95% of EPSCs were accepted, and their amplitudes and frequencies were further averaged.

To study the mEPSCs, neurons were recorded with a whole-cell patch electrode containing K gluconate (E_Cl-_ = -70mV) and were held at a potential of approximately -70mV (the E_reversal_ of IPSPs) by applying a small depolarization or hyperpolarization current.

AP threshold was calculated as the membrane voltage potential at which point the slope of the first (during positive current injection) was greater than 10 V/s.

### 5. Presynaptic Calcium Imaging

Fluorescent probe Ca Green-1 AM (Invitrogen), which exhibits an approximately 100-fold increase in emission intensity upon Ca^2+^ binding and reflects intracellular Ca^2+^concentration changes, was used for presynaptic Ca^2+^ measurements. Fluorescence measurements were performed 20 min after local injection of Ca Green-1 AM in the stratum radiatum as described previously [53, 54]. In brief, a small amount of dye is pressure-injected into the stratum radiatum using a Picospritzer II (General Valve, Fairfield, NJ) via a pipette of 2–3 μm tip diameter. Thirty minutes after injection, brain slices are illuminated at 506nm and separate images are taken in a small spot in the striatum radiatum area, 300–500 μm away from the injection site, to avoid contamination of the optical recordings by accidental postsynaptic indicator loading. Only slices with stable initial Ca Green fluorescence, confirmed after 20 min loading (about 60% of slices), were used. A BX51WI Olympus Spinning Disk Confocal microscope designed for simultaneous flourescence and electrophysiology experiments, equipped with 4 X (N.A 0.10) and 40 X water immersion (N.A 0.80) Olympus objectives was used. A digital EMCCD camera, (Cascade: 512B, 16-μm pixels, 512 x 512; Photometrics, Tucson, AZ, USA), monitored changes in Ca Green-1 fluorescence. A long pass filter block set XF104-2 (Omega Optical, excitation, 500 nm; emission, 545 nm and a dichroic mirror 525 nm) was used to visualize Calcium Green-1 fluorescence. Images were acquired every 1 min, stored and analyzed using Image-Pro Plus, (Media Cybernetics Inc., Silver Spring, MD, USA). All measurements were performed in the similar small (100 μm) regions of interest in the stratum radiatum. For statistical analysis, all fluorescence measurements were normalized to initial stable basal level in each slice.

### 6. Drug Preparation

Drug solutions were prepared fresh at the start of each experiment to prevent degradation by environmental factors e.g. light. Solutions were made using de-ionized water (pH 5–6, resistance 18.2 MΩcm) from a Milli-Q UV plus system.

ChTX was dissolved in water and stored in small aliquots at −20°C. It was then dissolved to its final concentration of 10 nM. The sodium channel blocker, tetrodotoxin (TTX, 1 μM), was used to block initiation and propagation of action potentials. The selective A1 –receptor antagonist, 8-cyclopentyltheophylline (8-CPT; Sigma, St. Louis, MO), was initially dissolved in DMSO and subsequently in ACSF to give a final concentration of 10 μM.

Bis-(*o*-aminophenoxy)ethane-*N*,*N*,*N*′,*N*′-tetraacetic acid acetoxymethyl ester (BAPTA-AM) and EGTA-AM (Molecular Probes, Eugene, OR) were initially dissolved in DMSO, and then diluted to their final concentrations in the ACSF. DMSO concentration in ACSF was 0.0001% for 1 μM BAPTA-AM and 0.00006% for 50 μM of EGTA-AM. The chelator freely entered the cell due to the AM moiety and was then deesterified to cell-impermeant BAPTA. Probenecid (Sigma, St. Louis, MO) was dissolved in 1 M NaOH and subsequently buffered with HCl acid to pH 7.4. Whenever probenecid was used (1 mM) care was taken to adjust the sodium concentration of the ACSF. Probenecid and DMSO did alone did not alter fEPSP at concentrations used.

### 7. Statistics

fEPSP amplitudes were given by pClamp software by measuring the maximum negative deflection from baseline. Data analysis was performed with Microsoft Excel custom Excel 2000 (Microsoft Corp., Redmond WA). Statistical significance was measured using paired/unpaired t-tests or ANOVA, as required. Whenever statistical tests were performed on normalized data, the latter was first arcsine transformed. Effects were considered statistically significant at *p* < 0.05.

## Results

### 1. OGD causes a time-dependent decrease in fEPSP amplitude

**Fig 1:**
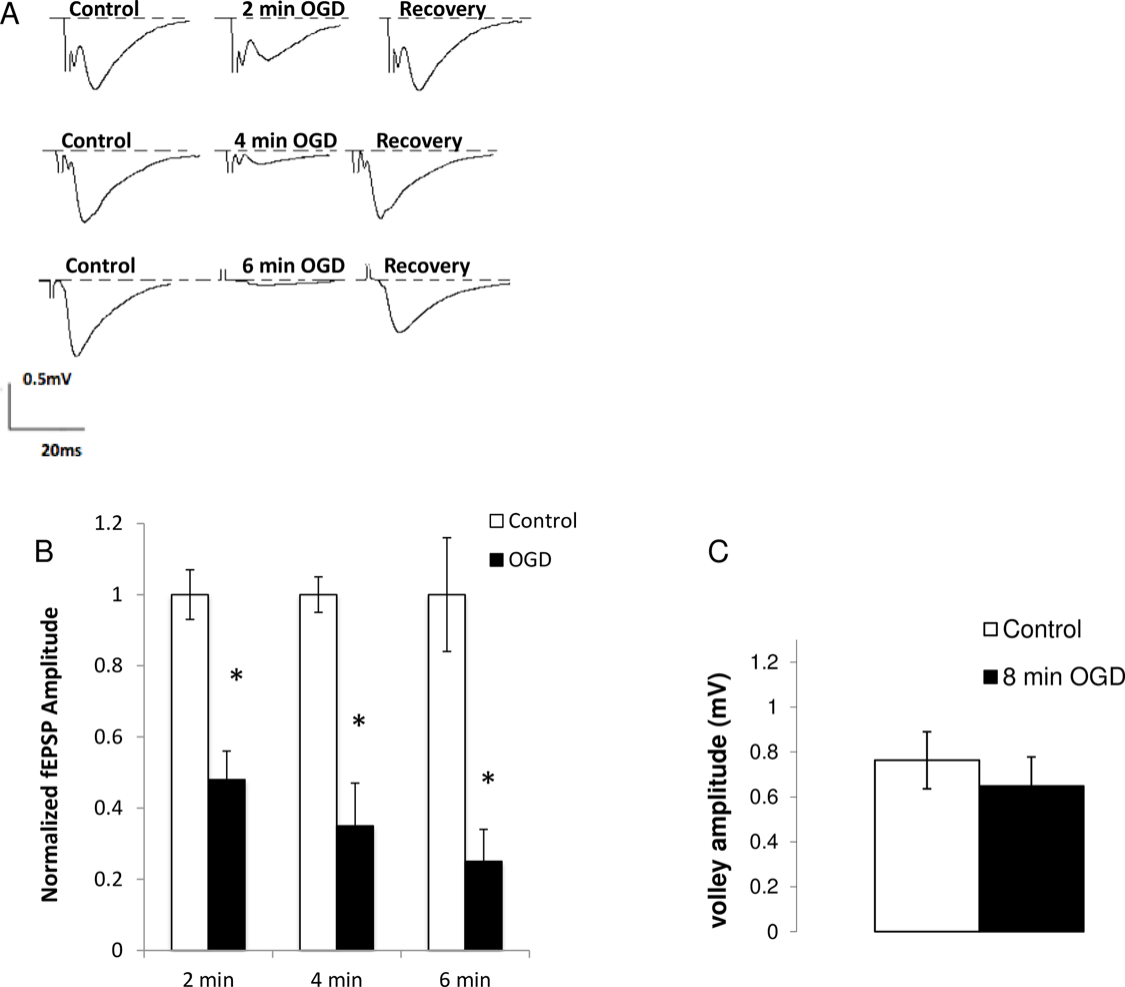
Ischemia depresses the amplitude of evoked synaptic transmission without changes in the presynaptic volley amplitude. (A). fEPSPs were recorded from the stratum radiatum in the CA1 region while the Schaffer collaterals were stimulated every 15 s. fEPEP amplitude decreased reversibly in a time-dependent manner after 2 min (n = 7), 4 min (n = 5), and 6 min (n = 5) of OGD. (B) fEPSP amplitudes produced by 2 min, 4 min, 6 min of *in vitro* ischemia relative to controls. (C) Fiber volley amplitude after 8 min of OGD (n = 6, p>0.05) Average plotted as mean ± SE. * p < 0.001: paired student t-test. OGD: Oxygen-glucose deprivation, fEPSP: field excitatory postsynaptic potentials.

Following 2, 4, and 6 min of OGD, the amplitude of fEPSPs diminished to 48 ± 8% (n = 7), 35 ± 12% (n = 5), 25 ± 9% (n = 5) of the original pre-ischemic value (*p*<0.05, Fig 1A and 1B). Is this depression in fEPSP related to reduced AP invasion of the presynaptic terminal or related purely to synaptic transmission? We measured the fiber volley size and observed no change in its amplitude for up to 8 min of ischemia (Fig 1C; *p* >0.05). These results suggest that OGD decreases evoked neurotransmitter release in a time-dependent manner without impairing presynaptic AP invasion, and confirm that our system produces results similar to those reported in other studies [6, 25].

### 2. OGD-induced depression of fEPSP is due to a presynaptic mechanism

**Fig 2:**
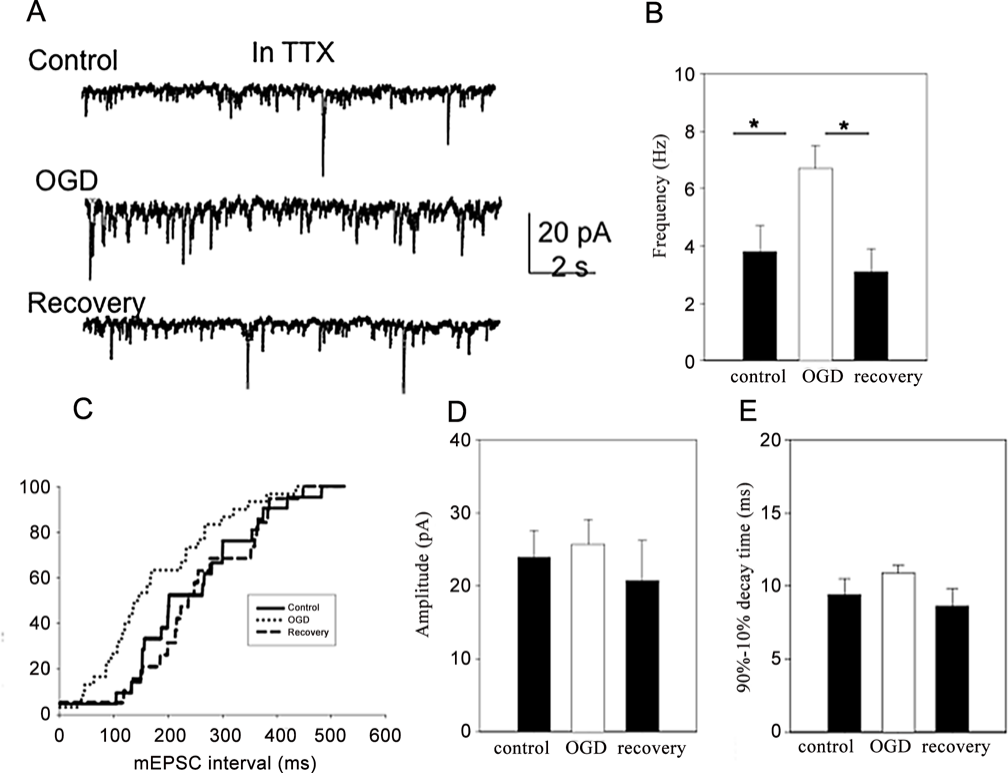
Effects of transient OGD on mEPSCs in CA1 neurons (n = 8). Experiments were performed in the presence of TTX (1.0 μM). (A) Voltage clamp recording from a CA1 pyramidal cell before, during transient ischemia, and 10 minutes after recovery. Note the transit inward currents, which represented mEPSCs, increase in frequency. (B) Frequency of mEPSCs increased during transient OGD. (C) Cumulative curve plot showing reduced intervals (increased frequencies) of the mEPSCs during OGD compared to control and recovery. (D) Amplitude of mEPSCs did not change in transient ischemia. (E) The decay time of mEPSC was not altered by the 2 min OGD.

To determine whether the depression of synaptic transmission is due to pre- or post- synaptic mechanisms, we recorded spontaneous miniature excitatory postsynaptic currents (mEPSCs) in the presence of TTX (1.0 μM) from CA1 neurons. It is generally recognized that the frequency of mEPSCs is mainly a presynaptic phenomenon [55, 56] and changes in their frequency reflect changes in the presynaptic terminal. Since APs are prevented by TTX, the mEPSCs are due to spontaneous quantal transmitter release. Fig 2A shows typical mEPSCs before, during 4 minutes of OGD and 10 minutes after OGD. Transient OGD induced an increase in the frequency of the mEPSCs (*p* < 0.05; Fig 2B) but the averaged amplitude of the mEPSC was unaltered (*p* > 0.05; Fig 2C). Additionally, mEPSCs decay time (90%-10%) was unaltered (9.4 ± 1.1 ms before, 10.9 ± 0.5 ms during OGD and 8.6 ± 1.2 ms after recovery; Fig 2D). These results suggest that the alteration of the fEPSP during transient OGD is likely due to a presynaptic mechanism [25].

**Fig 3:**
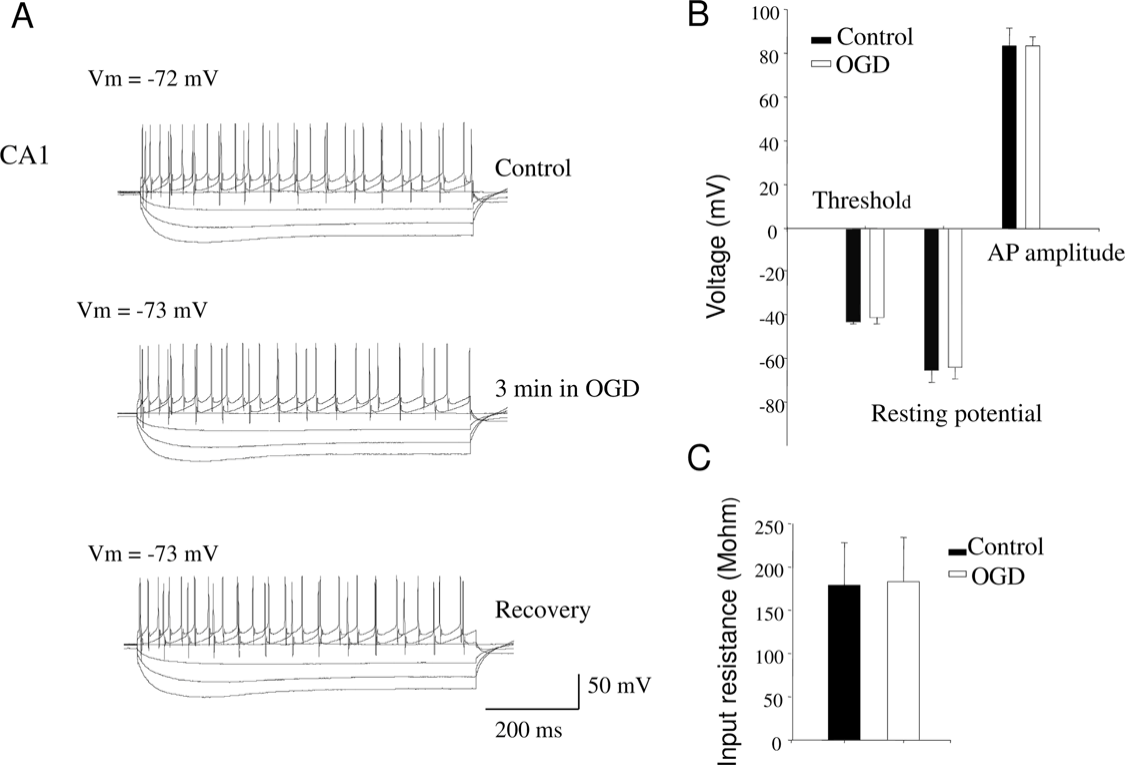
Intrinsic properties of the postsynaptic CA1 neurons did not change during transient OGD (n = 8). (A) Sample recording from a cell when constant current steps were applied via the patching electrode to quantify the spike and membrane properties from the hyperpolarizing and depolarizing voltage response. The current step protocol was used through all experiments when I-V curves were obtained. (B) 2–4 minutes of transient OGD did not cause significant changes in the transmembrane potential, threshold of firing action potential, and the size of AP. (C) 2–4 minutes of transient OGD did not cause significant changes in input resistance. * *p* < 0.05. mEPSC: miniature excitatory postsynaptic current

Is the change in fEPSP amplitude during transient ischemia a result of alterations in the intrinsic properties of the patched CA1 neurons? Responses to constant depolarizing and hyperpolarizing currents into the cell (Fig 3A) and I-V responses of the pyramidal cells before, 4 minute after the initiation of OGD and after 10 minutes of recovery were measured as well as resting membrane potential, threshold, and AP amplitude of the patched cells. No significant changes were observed in these measures (Fig 3B, G, *p* > 0.05). The resting potential was -64.3 ± 5.6 mV in control and -63.0 ± 5.3 mV during OGD; Threshold was -42.2 ± 1.1 mV in control and -40.3 ± 3.0 mV in OGD; AP amplitude was 83.4 ± 8.1 mV in control and 83.4 ± 4.1 mV in OGD. Although changes in input resistance can happen during longer (up to 6 minutes) OGD episodes [57, 58], we did not observe significance changes (178.8 ± 49.4 MΩ in control and 183.1 ± 51.1 MΩ in OGD, *p* > 0.05; Fig 3C). These results suggest that OGD-induced fEPSP depression is not likely due to alteration in the intrinsic properties of the postsynaptic neurons.

### 3. Some OGD-induced decrease in fEPSP persists in the presence of an A_1_-antagonist

**Fig 4:**
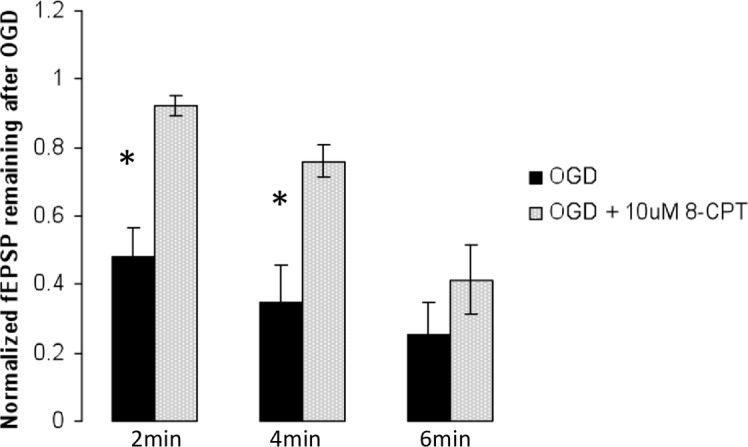
Effect of 10 μM 8-cyclopentyltheophylline (8-CPT) on evoked EPSP depression produced by *in vitro* ischemia. Some OGD-induced decrease in fEPSP persists in the presence of an A_1_-antagonist. 8-CPT reduced the OGD-induced percentage change in fEPSPs relative to control after 2 min (n = 5, *p*<0.0003) and 4 min (n = 5, *p*<0.005), but not after 6 min (n = 6, *p*>0.14) of OGD. * p < 0.05, Student t-test

To better quantitatively understand the role of adenosine on fEPSP, we exposed hippocampal slices to 8-CPT (10 μM), a potent A_1_-receptor antagonist. This significantly reduced the OGD-induced percentage change in fEPSPs relative to control after 2 and 4 minutes (*p*<0.0003, *p*<0.005, respectively; Fig 4), but not after 6 min of OGD (*p*>0.14). These results confirm that the mechanism of decreased evoked transmission during ischemia involves adenosine, at least during 2 and 4 min of ischemia. If adenosine was the only mechanism causing the changes in evoked release during ischemia, we would expect normalized fEPSP values of 1 in the group treated with 8-CPT. However, the fact that fEPSPs did still significantly decrease even in the presence of 8-CPT (10 μM), at least at 4 min (*p*<0.006) and 6 min (*p*<0.002) of OGD, suggest that other mechanisms contribute.

### 4. Decreased fEPSPs during OGD is correlated with increased presynaptic [Ca^2+^]_i_

**Fig 5:**
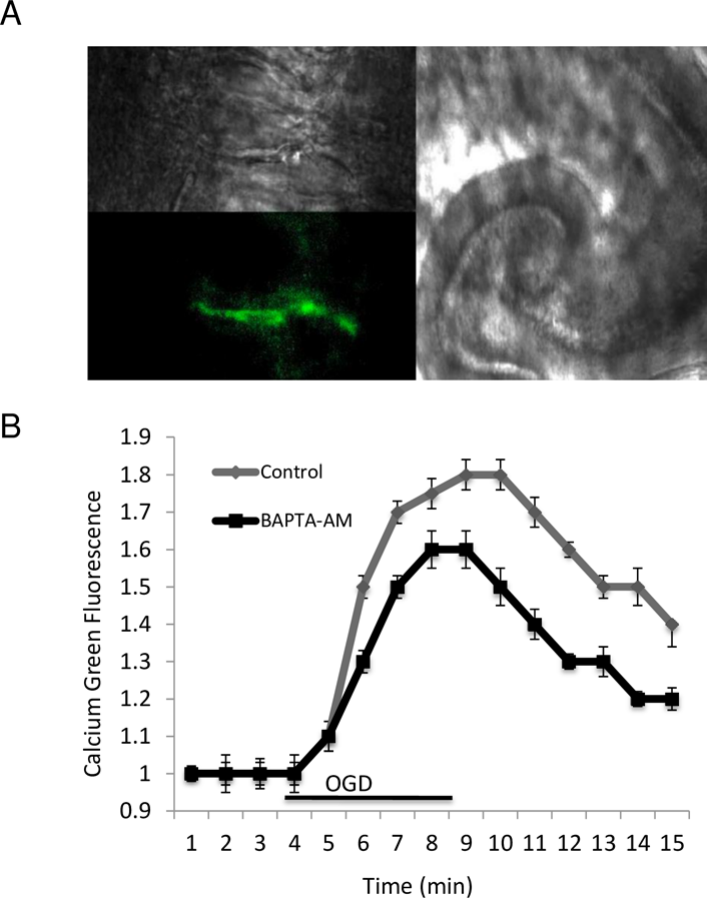
Presynaptic intracellular calcium fluorescence measurement from the CA1-stratum radiatum region using Ca Green-1. Calcium increases in the presynaptic terminal following OGD and is reduced by administration of cell- permeant calcium chelators (BAPTA-AM). (X40 N/A 0.8). A. High power images of axons locally loaded with Ca Green-1 (B) The effect of OGD on intracellular calcium in control (n = 5) and in the presence of 1 μM BAPTA-AM in presynaptic terminals (n = 6).

We have previously shown that there is no significant change in cytosolic calcium peaks after 30 min of BAPTA-AM perfusion [53]. OGD was administered after 4 min of stable basal Ca Green-1 fluorescence, reflecting intracellular level of calcium. We noticed a gradual increase of florescence, that directly reflects presynaptic [Ca^2+^]_i_ change, in the control group up to 1.82 ± 0.04 times the initial level, compared to an increase of 1.52 ± 0.03 (p<0.05) following prior BAPTA-AM perfusion (Fig 5A and 5B). After reperfusion, the Ca^2+^ signal slowly dropped, but did not return to the initial level within 10 min. These results suggest that the depression of fEPSPs during ischemia is associated with increases in presynaptic [Ca^2+^]_i_, a phenomenon that could potentially be partially prevented by the administration of a cell-permeant calcium chelator [53].

### 5. Buffering [Ca^2+^]_i_ during OGD reduces the ischemia-induced depression of fEPSPs

**Fig 6:**
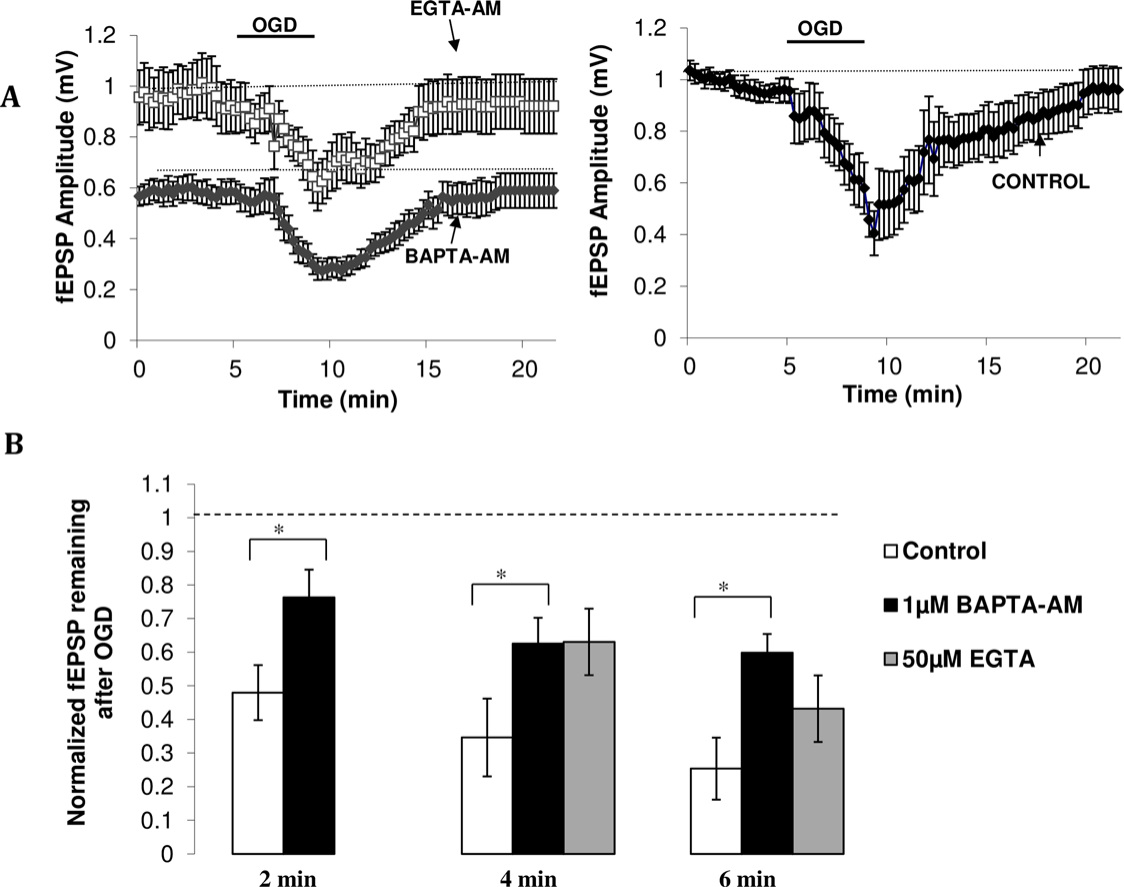
Cell-permeant calcium chelators reduce ischemia-induced depression of fEPSP amplitudes. (A) Time course of the depression and subsequent recovery of fEPSP amplitudes in drug (left) and control (right) condition. *Left* Calcium chelator data. 4min OGD in the presence EGTA-AM (grey triangle, n = 6) or BAPTA-AM (black circle, n = 6) leads to a smaller depression of fEPSP amplitudes relative to control, along with faster recovery of the response (approximately 5–6 min). *Right* Control data. 4min of OGD produces a large depression of fEPSP amplitude, which then takes approximately 9 min to recover (n = 5). (B) Amount of fEPSP amplitude remaining after oxygen-glucose deprivation. 1 μM BAPTA-AM increases the amount of evoked neurotransmission remaining after 2 min (n = 7), 4 min (n = 6) and 6 min (n = 5) of ischemia relative to control (n = 7, 5, 5, respectively). 50 μM EGTA-AM (n = 6) shows similar effects to BAPTA-AM (1 μM) at 4 min of OGD, with both chelators increasing the fEPSP amplitude remaining after OGD. At 6 min however, EGTA-AM does not significantly reduce OGD-induced depression of fEPSP amplitude relative to control (n = 6) Data plotted as mean ± SE. *p < 0.*05*, ANOVA.

Since the mechanism responsible for the ischemia-induced depression of fEPSPs is most likely presynaptic in origin and is correlated with increased presynaptic [Ca^2+^]_i_, we next asked if the increased [Ca^2+^]_i_ contributed to this phenomenon. If so, buffering [Ca^2+^]_i_ during ischemia should prevent some of the decrease in fEPSPs. BAPTA-AM prevented some of the OGD-induced depression in fEPSPs by increasing the percentage of fEPSP remaining after 2 min of OGD from 48 ± 8.% (n = 7) to 76 ± 8% (n = 7 see Fig 6B). The drug had similar affects at longer durations of ischemia (see Fig 6B). BAPTA-AM reduced basal fEPSP amplitudes and increased paired-pulse ratios (*p*<0.05), suggesting lowered quantal release (data not shown; [59]).

We next asked whether the effects of BAPTA-AM were due to the calcium chelator itself or due to a difference in initial fEPSP baseline. As previously shown [52, 60–62], BAPTA-AM reduces basal fEPSP amplitudes and so any changes during OGD were analyzed with reference to this new baseline. To test whether calcium chelators truly prevent some of the ischemia-induced changes in fEPSP amplitude, and to test chelators with different binding kinetics, OGD was administered in the presence of EGTA-AM, a chelator that did not reduce initial baseline amplitude [52]. The percentage of fEPSPs remaining after 4 min ischemia in the presence of EGTA-AM was 63 ± 10% (n = 6), greater than the control level of 35 ± 12% and analogous to the BAPTA-AM condition of 63 ± 8% (Fig 6A and 6B). These data suggest that calcium chelators reduce the ischemia-induced change in evoked neurotransmission. After 6 min of OGD however, only 43 ± 11% (n = 7) of the fEPSPs remained in the presence of EGTA-AM, compared to 60 ± 6% (n = 4) in the BAPTA-AM-pretreated condition, and 25 ± 9% (n = 5) in the control condition, suggesting that the faster binding kinetics of BAPTA-AM are important for the preservation of synaptic transmission in OGD.

### 6. Charydotoxin-sensitive channel blockade prevents the decrease in fEPSP

Having established that ischemia-induced depression of fEPSP amplitudes is related to increased presynaptic [Ca^2+^], we next asked what possible mechanisms could mediate this effect. One possibility is that increased [Ca^2+^]_i_ activates K channels, thus decreasing calcium influx through VGCCs, and neurotransmitter release. Thus, administration of a K channel blocker during ischemia should 1) widen the fiber volley and 2) prevent some of the ischemia-induced depression of fEPSPs.

**Fig 7:**
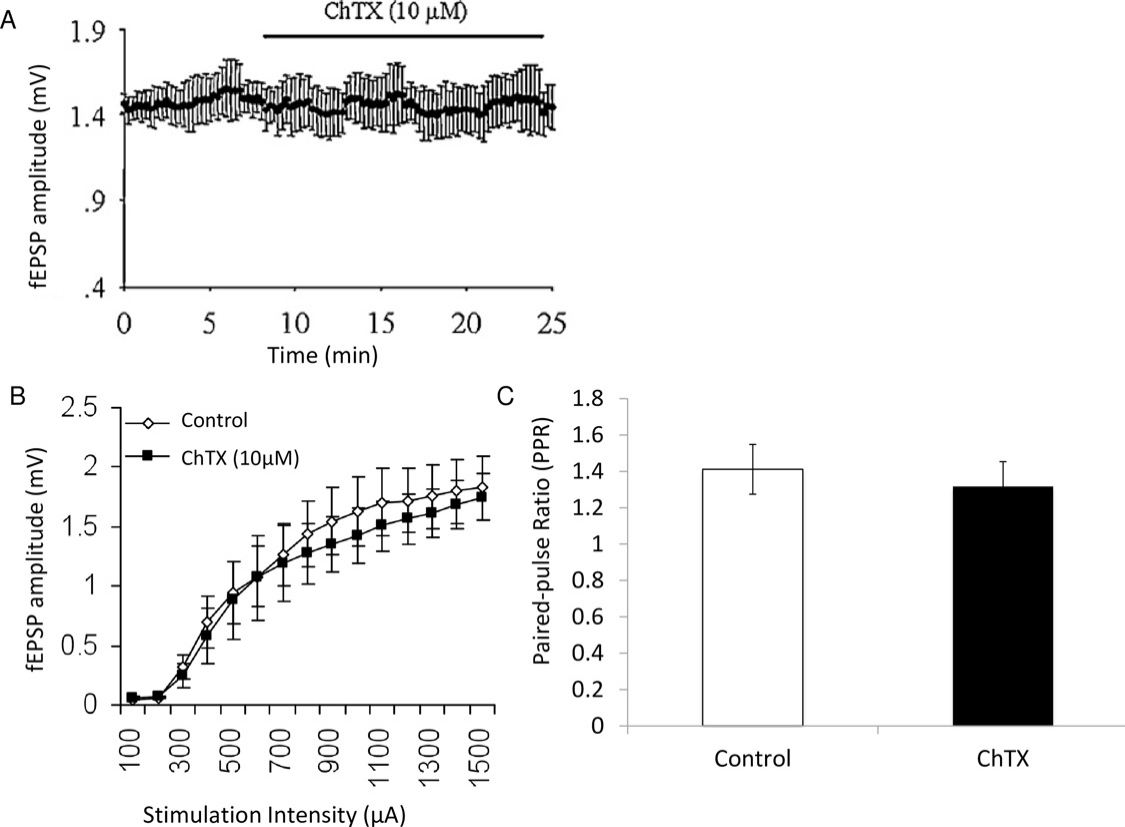
ChTX channel blocker does not change synaptic transmission under baseline experimental conditions. (A) fEPSP amplitude did not significantly change during application of ChTX (10 μM). (B) Input/Output curve for fEPSP amplitude versus stimulation intensity for control and ChTX (10 μM) condition. (C) Paired-pulse ratios were not significantly different in the control and ChTX (10 μM) condition. (p>0.05)

Administration of ChTX, which blocks BK and other K channels did not change baseline synaptic transmission (Fig 7A), a result consistent with work in rats [32] and mice [63]. Similarly no change was observed in the input-output curves or paired-pulse ratios before and after administration of 10 nM ChTX (Fig 7B and 7C). This suggests that, in this preparation, ChTX sensitive channels do not appear to regulate transmitter release under basal experimental condition.

**Fig 8:**
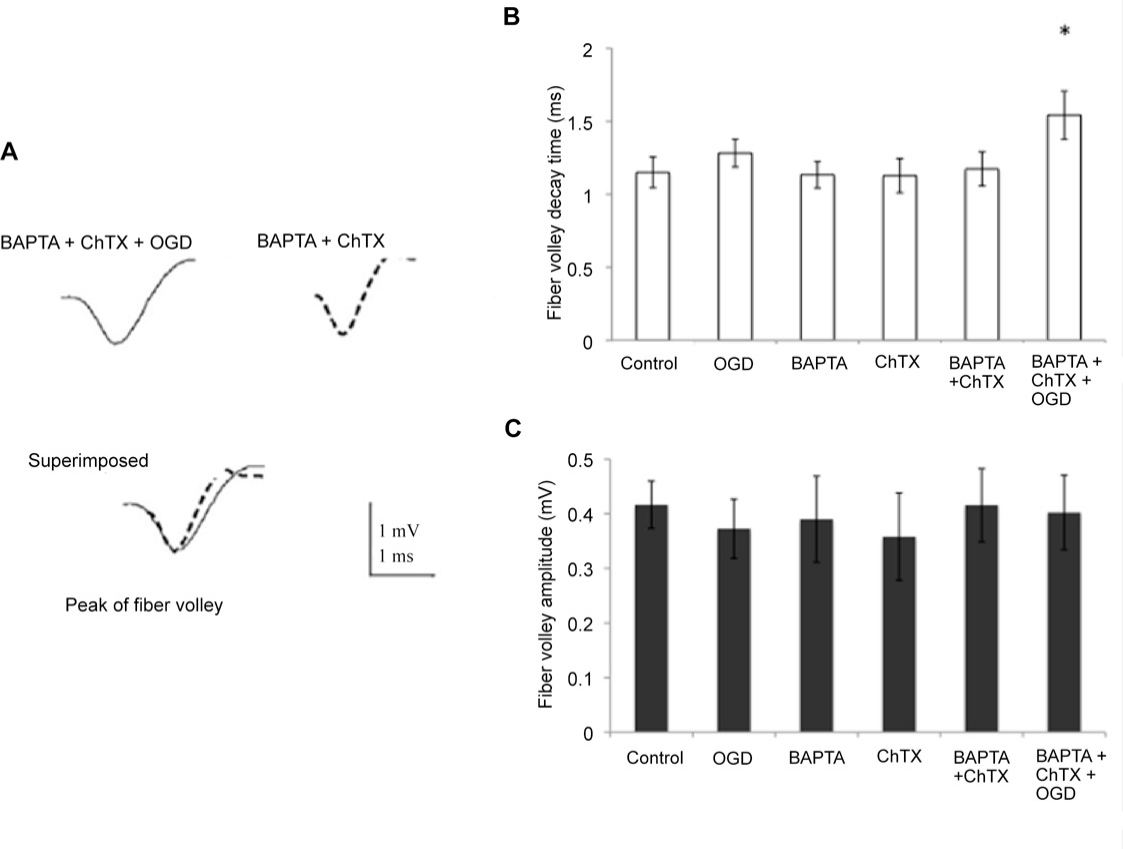
Decay time in the presynaptic component action potential (fiber volley) was lengthened in the presence of OGD and chelator + ChTX. (A) Fiber volley recorded in the presence of BAPTA-AM + ChTX + OGD (solid line) and in presence of BAPTA-AM + ChTX only (dotted line). Fiber volleys were aligned by their negative peaks and superimposed to compare their amplitude and decay time. (B) Decay time of the fiber volley was increased in BAPTA-AM + ChTX + OGD condition versus BAPTA-AM + ChTX alone. It remained unchanged in control, OGD, BAPTA-AM and ChTX condition. (C) Amplitude of the fiber volley was unchanged in the presence of BAPTA-AM + ChTX and BAPTA-AM + ChTX + OGD, as well as control, OGD, BAPTA-AM and ChTX conditions. * p < 0.05, Student t-test

Fiber volley in control, drug condition and the drug condition after 6 min of OGD in the same slice were analyzed by superimposing and aligning their negative peaks (for further details, see [63]). Their amplitude and decay time were then compared. Fig 8A shows the time course of the averaged fiber volley decay time in the drug condition and the drug condition and OGD combined. OGD alone, BAPTA, ChTX and the combination of the latter two did not change the fiber volley decay time (Fig 8B). However, after 6 min of OGD in the presence of BAPTA-AM and ChTX, fiber volley decay time was increased significantly relative to BAPTA and ChTX alone (1.17 ms ± 0.11 ms versus 1.54 ms ± 0.16 ms, Fig 8B, p < 0.03), suggesting that during OGD, the opening of voltage-gated calcium channels would be enhanced due to prolonged repolarization phase of the action potentials invading the presynaptic terminal. Blocking ChTX-sensitive channels subsequently widens the fiber volley during OGD. This finding implies that K+ channels, probably those triggered by raised intraterminal Ca, such as BK channels, narrow the presynaptic action potential by increasing the preterminal conductance and hyperpolarizing the preterminal membrane potential, thereby diminishing the action potential depolarization mediated Ca influx which triggers evoked neurotransmitter release. The fiber volley amplitude was unchanged throughout (see Fig 8C), suggesting no significant changes in the number of afferents activated by stimulation of the Schaffer collaterals.

**Fig 9:**
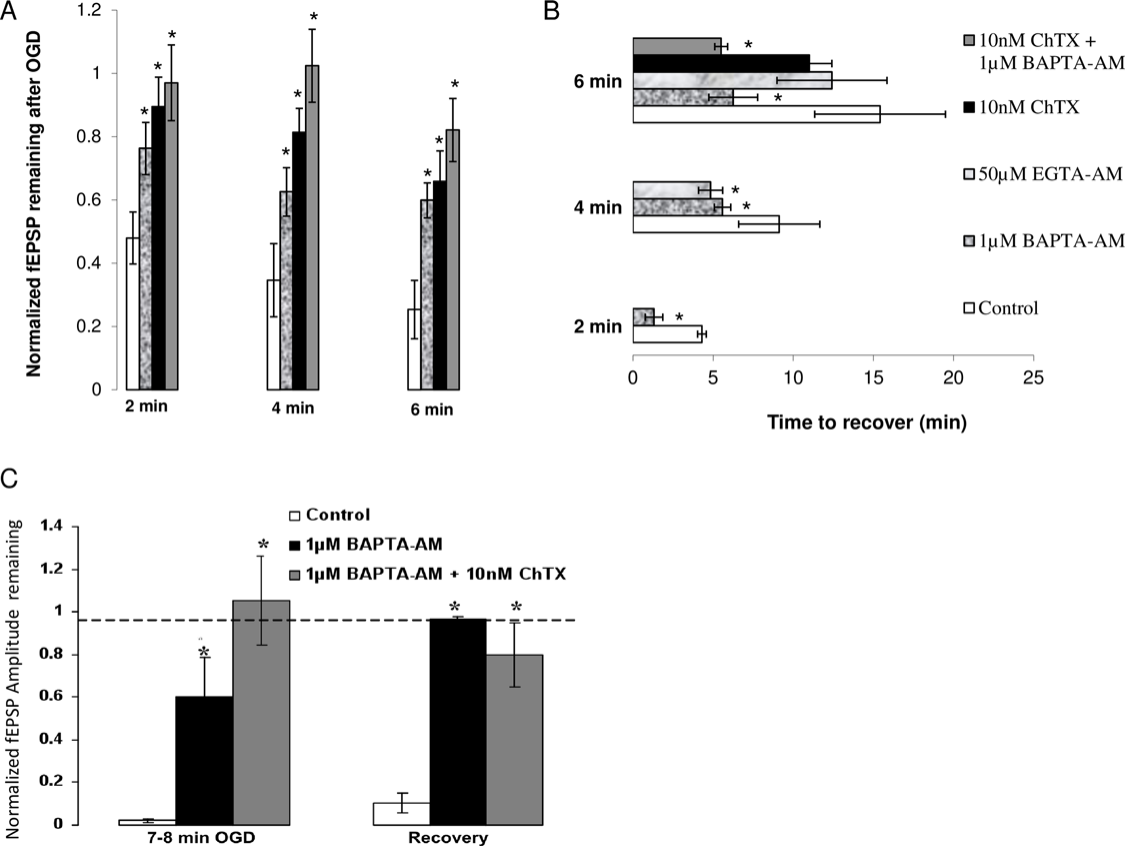
(A) Effect of calcium chelators and K channel antagonist, ChTX on fEPSP during OGD. 1μM BAPTA-AM increases the amount of evoked neurotransmission remaining after 2 min (n = 7), 4 min (n = 6) and 6 min (n = 5) of ischemia relative to control (n = 7, 5, 5, respectively). Similarly, the amplitude of fEPSPs remaining after 2 min, 4 min, 6 min of OGD (n = 6) is increased after administration of 10 nM ChTX. Combining ChTX and BAPTA-AM led to almost no change in fEPSP amplitude up to 6 min of OGD (n = 6). **(**B) Effect of calcium chelators, and BK channel antagonist on recovery of fEPSP after OGD. BAPTA-AM (1 μM) decreases recovery time from 2 min (n = 7), 4 min (n = 6) and 6 min (n = 3) of *in vitro* OGD compared to control (n = 7, 5, 5, respectively). EGTA-AM (50μM) shows similar effects to BAPTA-AM (1μM) by decreasing time needed for electrophysiological recovery after 4 min of OGD (n = 6) but not after 6 min (n = 7). ChTX (10 nM) did not significantly decrease recovery time after 6 min of ischemia (n = 4) but a combination of BAPTA-AM (1 μM) and ChTX(10 nM) significantly decreased recovery time after 6 min of OGD (n = 6). **(**C) BAPTA-AM and BAPTA-AM + ChTX promote increased tissue resistance to a long ischemic episode. BAPTA-AM (1 μM) increases the amount of fEPSP remaining after 8 min of OGD and leads to full recovery after 40 min of reperfusion post ischemia (n = 3) when control tissue has surpassed the point of functional recovery (n = 4). A combination of BAPTA-AM (1 μM) and ChTX (10 nM) leads to almost no change in fEPSP amplitude after prolonged OGD and leads to almost full recovery after 40 min of reperfusion (n = 5). Data plotted as mean ± SE. *p < 0.*05*, ANOVA, all relative to control condition.

Administration of 10 nM ChTX during ischemia showed that significantly more fEPSP remained after OGD in the ChTX condition compared to the control condition (*p*<0.05, see Fig 9A). These data suggest that activation of BK channels during OGD contributes to the depression of fEPSPs.

We next asked if pre-treatment with calcium chelators would promote quicker electrophysiological recovery after OGD. Cytosolic Ca^2+^ accumulation activates a complex cascade of events that lead to neuronal damage and cell death [64] and enhancing the cells’ calcium handling properties should render some protection. To test this, we looked at fEPSP recovery time after ischemia, defined by time required for fEPSP amplitude to return to its original value and remain there.

Results showed that calcium chelators reduced recovery time following 2, 4, 6 min of ischemia (Fig 9B). After 2 min of OGD, control tissue recovered after 4.1 ± 0.6 min (n = 7), while BAPTA-AM pre-treated tissue recovered after 1.54 ± 0.5 min (n = 7; *p*<0.004). Similar effects were observed after 4 min of ischemia in both BAPTA-AM and EGTA-AM- treated tissue. Tissue exposed to 6 min of OGD required 15.8 ± 1.4 min to recuperate (n = 4), with one slice failing to completely recover after 45 min of reperfusion, while BAPTA-AM-treated tissues achieved full recovery after 6.0 ± 1.3 min (n = 4). Slices exposed to 6 min of OGD in the presence of EGTA-AM recovered fully but the time required for recovery (12.4 ± 3.4 min, n = 7) did not significantly vary from control tissue.

Because the effect of ChTX on depression of evoked release during OGD was similar to the effect of BAPTA-AM, we wondered if ChTX would also promote faster electrophysiological recovery via a different mechanism. The data however showed that recovery time from 6 min of OGD was not decreased in the presence of 10 nM ChTX (n = 4; 11 ± 1.4 min) compared to control (n = 4; 15.8 ± 1.4 min, *p* = 0.24, see Fig 9B) with one slice failing to recover in the ChTX condition, similar to control. Collectively, results indicate that, although BK channel activation partially mediates ischemia-induced depression of evoked synaptic transmission, post-ischemic recovery of fEPSP is not solely dependent on ischemic activation of these channels but rather on the preceding increase in intracellular calcium.

### 7. Effects of BAPTA-AM and ChTX are not additive

Having determined that ChTX diminishes ischemia-induced depression of fEPSPs, we asked if BK channel activation is caused by the increased [Ca^2+^]_i_ that occurs during OGD. As discussed previously, ChTX does not affect basal neurotransmission. The fact that the drug results in a change in fEPSP amplitude during ischemia relative to control must mean that an event during ischemia renders this drug “effective”. An obvious candidate is the increased [Ca^2+^]_i_ that occurs during OGD. To see whether BK channels activation during OGD, and subsequent effect of ChTX, is due to increased [Ca^2+^]_i_, we combined ChTX and BAPTA-AM and measured fEPSP during ischemia. If increased [Ca^2+^]_i_ is responsible for the effects of ChTX, then the fEPSPs remaining after the combined condition should not be significantly different compared to the ChTX-alone condition. This is because BAPTA-AM would have already lowered calcium levels, thus making the channels less active and adding the ChTX would have no additional effects.

Results showed that, as expected, the amount of fEPSPs remaining after 2, 4, 6 min of OGD in the BAPTA-AM + ChTX condition were not significantly different from the ChTX alone condition (see Fig 9A). After 2 min of OGD, 96 ± 12% (n = 5) fEPSPs remained in the combination condition compared to 89 ± 9% (n = 6), in the ChTX alone condition (*p* = 0.32). Similarly, 102 ± 12%, and 84 ± 12% of fEPSPs remained after 4 min, 6 min of OGD, respectively, in the combination condition, compared to 81 ± 12% and 66 ± 10% in the ChTX alone condition (*p* = 0.15, *p* = 0.14, respectively). Together, these results suggest that activation of BK during OGD is likely dependent on the increase in intracellular calcium.

### 8. Application of BAPTA-AM and BAPTA-AM + ChTX delayed the critical point

We next asked if the buffering [Ca^2+^]_i_ alone or in combination with ChTX promoted recovery after prolonged OGD after which the cell does not normally functionally recover (critical point). We found that the critical time point after which the depression of fEPSPs was irreversible to be approximately 7–8 min. Results indicated that, in tissue treated with 1 μM BAPTA-AM and 1 μM BAPTA-AM + 10 nM ChTX, the amount fEPSPs remaining after 7 min of OGD was 56 ± 22% (n = 3; *p*<0.03) and 105 ± 21% (n = 5, *p*<0.01), respectively (see Fig 9C). This is significantly greater than in control tissue, in which the amount of fEPSPs remaining was only 2 ± 1% (n = 4). Additionally, treated tissue recovered almost fully to baseline fEPSP amplitude after a prolonged OGD, whereas control tissue did not. (see Fig 9C). Together, these results imply that increasing cells’ calcium handling capabilities promotes increased tissue resilience to a prolonged ischemic episode.

## Discussion

To our knowledge this study provides the first evidence that increasing the cell's calcium buffering capabilities with the use of cell-permeant calcium chelators can partially prevent depression of evoked neurotransmission during OGD in CA 1 neurons of the hippocampus, improve electrophysiological recovery and delay ischemic depolarization. Additionally, we show that OGD-induced increases in [Ca^2+^]_i_ contribute to the activation of BK channels, which in turn, partially mediates the depression of evoked neurotransmitter release. These findings add to the complex number of interplaying factors that mediate changes in synaptic transmission and neurotoxicity during OGD.

### 1. Increased [Ca^2+^]_i_ and ischemia-induced depression of evoked release

Our results show that OGD leads to a time-dependent depression of evoked neurotransmission, which is partly mediated by activation of the A_1_ receptors. This depression is likely presynaptic in origin, a concept that is in agreement with previous work, which have reported intact response of the postsynaptic neuron upon direct glutamate application both *in vitro* and *in vivo* during ischemia [25,65, 66].

Our study and work by others [25, 67] implicates a possible role for intracellular Ca^2+^in modulating the effects of ischemia on evoked neurotransmission. How does increased [Ca^2+^]_i_ lead to a depression of evoked release? Calcium is a key second messenger and its concentration is kept at approximately 100 nM in the cytosol, compared to approximately 1 mM in the extracellular environment [30]. The complex effect of increased [Ca^2+^]_i_ on synaptic release is therefore largely dependent on its transient microdomain localization. It is therefore possible that by loading tissue with a calcium buffer, we increased Ca^2+^ mobility [60, 68] so that microdomain concentrations never reach high enough levels to inactivate VGCCs responsible for evoked neurotransmission. Secondly, increased spontaneous release during ischemia was shown to result from Ca^2+^ mobilization from dantrolene-sensitive intracellular stores [69], This increased AP-independent release will most likely affect AP-dependent release [58]; because vesicles are drawn from the same limited pool [70] and depend on the same release machinery [71], there might be fewer docked vesicles ready for synchronized release by Ca^2+^ during an AP. A third possibility is through activation of BK channels.

### 2. Role of BK channels

BK channels are located in the presynaptic terminals of CA1 glutamatergic neurons [32, 72] and have been shown to mediate the fast phase of the afterhypepolarization [73], thus contributing to the repolarization phase of the AP. Previous work on the frog NMJ showed that BK channel blockers increase synaptic transmission in control conditions [74, 75], but mammalian studies have shown these channels to only be activated under "pathological" conditions of excessive depolarization and intracellular calcium accumulation [32, 63]. This however seems to be synapse/site-dependent, since BK channels were found to modulate evoked neurotransmitter release under basal experimental conditions in rat CA3-CA3 synapses [33].

We show that ChTX partially blocks the ischemia-induced depression of evoked release, and that this is possibly mediated by increased presynaptic [Ca^2+^]_i_. It was recently shown that calcium chelation with BAPTA prevented the BK channel-mediated excessive neurotransmitter release that is seen in the CA pyramidal neurons of a mouse model with fragile X syndrome, and that this is secondary to disease-mediated impairment of BK channel sensitivity to calcium [41]. Previous studies [32] have suggested that BK channels may also play a key role in ischemia, as the depolarization-induced spike broadening activates these channels, which then take over the repolarization phase of AP. However our results must be tempered with the fact that ChTX can also inhibit several other types of voltage-gated K+ channels, such as Kv1.3, with nanomolar affinity. Hence our study does not confirm this previous hypothesis, but does implicate a role for K+ channels in the OGD-induced depression of synaptic transmission. EGTA is a slow binding chelator which, unlike BAPTA, does not alter the microdomain calcium signal enough to reduce basal synaptic transmission. So how did EGTA become "effective" after 4 min of ischemia? One possibility is that, because OGD also causes a global cytoplasmic calcium rise, as well microdomain increases, this bulk calcium also contributes the OGD-induced synaptic depression. This removes the time critical element as the chelator no longer has to trap the Ca^2+^ before it can diffuse for example, 10 nm to a nearby channel [74, 75].

Why does the effectiveness of EGTA disappear at longer durations of ischemia? EGTA’s lower binding kinetics, when compared to BAPTA, makes it a less effective buffer at higher calcium concentrations [60, 61]. It is therefore possible that in our preparation, global calcium increases to a critical value between 4–6 min of ischemia, so that the buffering capabilities EGTA are limited by its binding speed. Additionally, the acidosis that occurs during OGD decreases the calcium affinity of EGTA-AM, thus reducing its effectiveness as a buffering agent [60, 76].

How do we know where (presynaptic vs postsynaptic vs both) the calcium chelators and BK channels were acting? Even though BAPTA application is causing a global buffering of calcium, we think that it is unlikely that its postsynaptic effects are mediating the observed results. We have previously investigated the postsynaptic effect of BAPTA-AM [77] whereby we injected the salt directly into single cells with a recording electrode. Interestingly, both EPSPs and IPSPs were increased rather than decreased. This could be related to the chelator’s interference with calcium-dependent inactivation of transmitter-gated channels that mediate the postsynaptic response. Furthermore, administration of BAPTA-AM in control slices led to an increase paired-pulse ratio, suggesting lowered quantal release [59]. This could be because chelation of the calcium by BAPTA-AM causes decreased vesicular exocytosis with the first pulse, allowing more vesicles to be released with the second pulse. This results in the second pulse having larger amplitude relative to the first, and increases the ratio, supporting a presynaptic effect.

### 3. Role of calcium chelators and a K channel antagonist on recovery time

If both BAPTA-AM and ChTX prevent ischemia-induced depression of fEPSPs, why does BAPTA-AM promote electrophysiological recovery while ChTX does not? The ability of calcium chelators to promote faster functional recovery of the synapse after ischemia implies that they may be protective. However, the depression of evoked neurotransmission is thought to be a protective mechanism and so preventing it could lead to glutamate excitotoxity and cell death (13). In fact, compounds such as A_1_ receptor agonists, which enhance this ischemia-induced depression, have been suggested for neuroprotection [78]. Excitotoxicity is heavily calcium-dependent and by exogenously providing both the pre and postsynaptic cell with a calcium buffer at a time when its endogenous buffering system is probably overwhelmed, we may be thwarting microdomain accumulation of excitotoxicity's chief perpetrator. There exists many studies that support the association between calcium overload and neurotoxicity [79–82] and much evidence suggest that the mechanisms by which chelators provide neuroprotection are considerably complex [61, 83, 84]

Previous work has shown that the release-enhancing effect of BK channels antagonists in the frog NMJ is prevented when a membrane-permeant calcium chelator, such as BAPTA-AM, is introduced [74, 75] This suggests that the effect of the activity of the channels, and the subsequent effect of ChTX, if its main action is in fact on BK channels in these experiments, is dependent on intracellular calcium. Our data are in agreement with this concept, as the effects of the drugs were not additive. Although the effects of both drugs on the depression of evoked release are similar, chelators reduce recovery time through their ability to control calcium, something that ChTX did not do, indicating that blocking K+ channels is probably not protective against acute ischemia. In fact, some K channel blockers would slow repolarization, thus increasing excitability and worsening neurotoxicity.

### 4. Role of calcium chelators in establishing the "critical point"

Exposure of fura 2-loaded neurons to high concentrations of glutamate cause a brief increase in intracellular calcium followed by a decline as compensatory buffering mechanisms in the cells which are recruited [85]. There is then a large second increase in calcium, which denotes irreversible deregulation of calcium homeostasis [86]. This depolarization, which is “spreading depression-like”, is irreversible and follows the disappearance of synaptic transmission [87]. It signifies the critical point, after which cell death is imminent, although some studies suggest that the late calcium signal is not necessarily the mediating factor of the neurotoxicity, as it is the source of the calcium rather than the total calcium load that mediates cell death [85, 88]. Therefore, depending on the calcium source, cascades mediating cell death may be activated during this initial, transient calcium signal, and the secondary calcium signal may be merely a consequence of the initial calcium disturbance [86]. Our results show that application of BAPTA-AM delayed the critical point. Providing an exogenous buffering system to the cell (i.e. BAPTA-AM) helped maintain calcium homeostasis for longer, delaying the onset of irreversible cell death.

We acknowledge that our study suffers from some limitations. Firstly, the preparation limits the ability to identify the cell types that contributed to the measured responses. OGD likely influences adjacent neurons and glia, which in turn contribute to the neuronal population responses used for recordings. The important role of glia signaling cannot be ignored. It is now known that ischemia-induced increases in intracellular calcium triggers the release of ATP from astrocytes [89, 90]. This is rapidly hydrolysed to adenosine in the extracellular space and contributes to the depression of EPSCs [91]. One alternative hypothesis therefore is that calcium chelators decrease the ischemia-induced depression of evoked transmitter release by decreasing calcium-induced ATP release. Although a fair possibility, it is important to acknowledge that ATP can also cause glutamate release from the astrocytes through activation of P2X receptors [92]. The contribution of astrocytes to increasing overall excitability or inhibitory tone is therefore arguable.

In conclusion, we demonstrate that ischemia-mediated increase in Ca^2+^_i_ in the presynaptic terminal blocks calcium-mediated, AP-dependent excitatory neurotransmitter release, in part through activation of BK channels, and that chelation of raised Ca^2+^_i_ hastens the post-ischemic recovery of the evoked neurotransmitter release. These findings suggest new therapeutic strategies for improving impaired synaptic transmission following brief ischemia.
